# Real-Time Monitoring of Chemisorption of Antibodies onto Self-Assembled Monolayers Deposited on Gold Electrodes Using Electrochemical Impedance Spectroscopy

**DOI:** 10.1021/acs.langmuir.5c01062

**Published:** 2025-06-17

**Authors:** Soraia Oliveira, Brian V. Jones, Pedro Estrela, Paulo R.F. Rocha, Nuno Miguel Reis

**Affiliations:** † Department of Chemical Engineering, 1555 University of Bath, Claverton Down, Bath BA2 7AY, United Kingdom; ‡ Centre for Bioengineering & Biomedical Technologies (CBio), University of Bath, Claverton Down, Bath BA2 7AY, United Kingdom; § Department of Life Sciences, University of Bath, Claverton Down, Bath BA2 7AY, United Kingdom; ∥ Department of Electronic & Electrical Engineering, University of Bath, Claverton Down, Bath BA2 7AY, United Kingdom; ⊥ Centre for Functional Ecology-Science for People & the Planet, Associate Laboratory TERRA, Department of Life Sciences, 37829 University of Coimbra, Coimbra 3000−456, Portugal; # Centre of Biological Engineering (CEB), University of Minho Campus de Gualtar, Braga 4710-057, Portugal

## Abstract

Understanding protein binding to biosensing surfaces is paramount to the design and performance of biosensing devices in fields such as point-of-care testing and bioanalytics. Here, we systematically demonstrated the use of electrical impedance spectroscopy (EIS) and equivalent circuit modeling for real-time tracking of chemisorption of IgG antibody to large-area circular gold electrodes (1.3 mm^2^) functionalized with a self-assembled monolayer (SAM). Using 1 μg/mL IgG and 5 mM of [Fe­(CN)_6_]^3–/4–^, the measured low-frequency impedance proved sensitive to both equilibrium and kinetics of antibody binding, with a slope of ∼74 kΩ/h for the first 2 h and taking approximately 4 h to reach equilibrium in a standard 6 mm-diameter well. Changes in impedance were found to be proportional to the reciprocal of the change in capacitance up to half-to-full IgG monolayer bound to the SAM. Further experiments with a flat microchannel confirmed that the low-frequency impedance and equivalent charge-transfer resistance (*R*_ct_) depend not only on antibody diffusion but also on the surface-to-volume ratio, which can represent a major challenge previously unreported for the miniaturization of EIS in microfluidic devices. This challenge arises as it requires a higher concentration of [Fe­(CN)_6_]^3–/4–^, of 50 mM or above, which was found to interfere with *R*_ct_ during chemisorption at low IgG concentrations. Chemisorption of IgG to SAM was confirmed with fluorescence microscopy and FTIR. This study marks, to the best of our knowledge, the first experimental demonstration of EIS as a real-time technique for quantitation of Langmuir isotherms during chemisorption of antibodies to SAM, with the potential to improve the design of EIS-based biosensors, especially those integrated into microfluidic devices.

## Graphical abstract

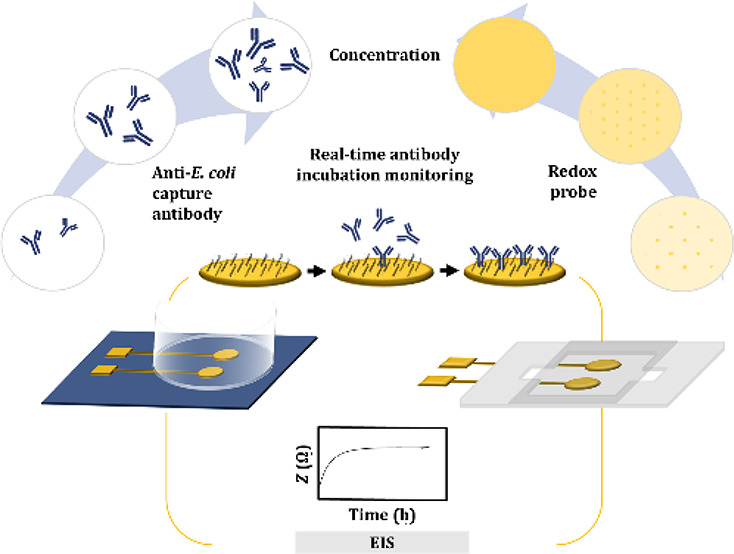

## Introduction

A shared goal in diverse fields, such as the development of biosensors and pharmaceuticals, is to better understand and optimize protein immobilization. A simple method to perform the immobilization of proteins onto solid substrates is by passive adsorption; however, it often comes with its own limitations, including limited surface area for binding, poor stability of the antibody layer, an extended time scale required for equilibrium, and the need to control antibody orientation—a vital step in diagnostic test manufacturing.^1^ Consequently, adsorption is often avoided as an immobilization strategy. The development of reliable and sensitive immunoassays relies on the ability to understand stability, density, orientation, and distribution (antibody monolayer or half-monolayer), regardless of the immobilization strategy used.^1–3^ Measuring real-time binding events is paramount to the development of any biosensing device involving a biofunctionalized surface, as both orientation and the extent of binding impact the performance of the biosensor.^1^ Yet, so far, this has been restricted to advanced optical techniques like surface plasmon resonance (SPR). Electrochemical biosensors are emerging in both biomedical and environmental domains due to their numerous advantages, including rapid response times, low cost, mass production feasibility, and the potential to be integrated with miniaturized electronics and devices.^4^ In particular, sensors based on Electrochemical Impedance Spectroscopy (EIS) stand out for their ability to enable label-free detection with high sensitivity,^5,6^ which is particularly attractive for numerous applications in biomedical testing and environmental monitoring.^7^ Studies have identified biosensing molecules on gold, with sensitivity being dependent on the gold surface and the immobilization of antibodies, with self-assembled monolayers (SAM) being frequently used in the functionalization process. However, little attention has been given to date to the thermodynamics or kinetics of binding of antibodies to EIS-based electrodes.

Small-signal EIS is an electrochemical method valuable for extracting comprehensive information related to various physical and chemical processes by determining their characteristic frequency response. Through EIS and equivalent circuit modeling, it is possible to determine the electrical properties of the electrode/electrolyte interface, offering insights into a variety of physical and chemical processes that occur on different time scales.^8–10^ In a conventional Faradaic impedance measurement, the interface is typically characterized by three key parameters: solution resistance (*R*_s_), double-layer capacitance (*C*_dl_), and charge-transfer resistance (*R*_ct_). *R*_s_, *C*_dl_, and *R*_ct_ are commonly modeled and quantified using the Randles equivalent circuit. Impedimetric sensors, depending on the nature of the transducing signal, can be categorized as capacitive, where the electrode surface is covered by a dielectric layer exhibiting no discernible Faradaic activity, or as Faradaic, when the electrode-confined film is either partial or conductive, allowing for the exchange of electrons with a solution-phase redox probe.^10,11^ Faradaic sensors—the most common—are based on the measurement of the charge-transfer resistance (*R*_ct_), which typically experiences a steric or electrostatic increase with immunorecognition events.^11,12^

Electroanalytical techniques present a promising avenue for achieving miniaturization and integration, demonstrating an adaptability that aligns with the requirements of biomedical diagnostics for point-of-care testing.^10,11,13^ For example, biosensor-based detection techniques offer an intriguing alternative for conducting straightforward, sensitive, rapid, selective, and cost-effective measurements for biomarkers and pathogens. Previous studies with pathogenic bacteria, such as , have also demonstrated the potential for real-time monitoring.^4,14^ Biosensors relying on the highly specific interactions between antibodies and antigens emerge as one of the most sensitive and specific surfaces for these applications.^15,16^ Consequently, we hypothesized that EIS could be applied for measuring real-time binding of antibodies onto gold EIS electrodes. A detailed review of the literature sugested there is a practice among the EIS biosensing community of carrying out EIS measurements as an endpoint.

Here, we present a systematic study of EIS as an alternative technique for real-time tracking of the binding of antibodies to a self-assembled monolayer (SAM) of mercaptohexadecanoic acid functionalized on the gold surface of electrodes. Our work specifically focuses on characterizing the chemisorption of antibodies onto the electrode surface, which is a critical step in the fabrication of electrochemical biosensors. While our findings may offer insights relevant to sensor applications, the primary aim was to establish EIS as a robust tool for real-time surface characterization rather than to assess molecular specificity or sensor performance in a complex matrix. Thus, this methodology will help speed up surface characterization and the development of EIS-based biosensors, including those designed for the detection of bacterial pathogens at the point-of care. Unlike other endpoint assays, such as the enzyme-linked immunosorbent assay (ELISA), our real-time approach allows for the determination of rate constants for adsorption. This provides crucial information about the efficiency and mechanism of the immobilization process, which is often overlooked. Understanding these kinetics is essential for optimizing immobilization protocols and predicting long-term sensor stability. However, we recognize that many sensor developers prioritize maximizing antibody loading on surfaces, sometimes without the use of SAMs, to enhance assay performance. While high-density immobilization is a valid strategy, it is often linked to steric hindrance and overlooks crucial aspects of antibody-surface interactions that can significantly impact sensor performance and reliability. Additionally, 50% of a full monolayer has been recommended to optimize performance and avoid steric hindrance.^1^ Our work explicitly focused on SAM-modified electrodes, as they provide a controlled environment for studying antibody adsorption. The SAM provides a consistent and reproducible surface for antibody adsorption, and by using a well-characterized SAM, we can more confidently attribute changes in EIS parameters to the adsorption of antibodies. Additionally, this work addresses how different buffer conditions affect the rate of antibody adsorption and the resulting interfacial properties. The knowledge gained about optimal immobilization conditions can be used to design better assays, even in the 96-well plate format. For example, understanding the kinetics could lead to shorter, more efficient immobilization protocols. Moreover, the principles and methodology can be generalized to study the adsorption of other biomolecules (e.g., proteins, DNA, aptamers) and to investigate the influence of different SAM chemistries, surface modifications, and solution conditions on the immobilization process.

## Experimental Section

### Solutions and Reagents

Rabbit polyclonal anti- (PA1–7213), used as the primary antibody, was purchased from Fisher Scientific (Rockford, IL, USA), and Goat anti-Rabbit IgG (H+L) Secondary Antibody (FITC) was obtained from Life Technologies (Paisley, UK). Surface functionalization of the gold electrodes was performed using 16-mercaptohexadecanoic acid (MHDA), *N*-(3-(dimethylamino)­propyl)-N′-ethylcarbodiimide hydrochloride (EDC), N,N-diisopropylethylamine (DIEA), and phosphate-buffered saline (PBS), all sourced from Sigma-Aldrich (Dorset, UK), and 2,3,4,5,6-pentafluorophenol (PFP), supplied by Fisher Scientific (Loughborough, UK). The primary experimental buffers were PBS and, as a measurement buffer, PBS with Potassium hexacyanoferrate­(III) (K_3_[Fe­(CN)_6_]), obtained from Fisher Scientific (Loughborough, UK), and Potassium hexacyanoferrate­(II) trihydrate (K_4_Fe­(CN)_6_) from Sigma-Aldrich (Dorset, UK). For washings, absolute ethanol and PBS with 0.05% (v/v) Tween-20 (Sigma-Aldrich, Dorset, UK) were used. SuperBlock was purchased from ThermoFisher Scientific (Lutterworth, UK). For the electrode cleaning process, acetone and 2-propanol, purchased from Sigma-Aldrich (Dorset, UK), were used. Phosphate-buffered saline was used at standard working (1×) concentration (137 mM NaCl, 2.7 mM KCl, 10 mM phosphate buffer, pH 7.4) and is hereafter referred to as “1× PBS” unless otherwise indicated.

### Sensor Fabrication and Cleaning

All electrochemical measurements were carried out using a two-electrode configuration, where the current-carrying electrodes were also used for sensing measurements.^5,6,17–19^ Each rounded-shaped electrode had an area of 1.3 mm^2^ and an interelectrode distance of 2 mm, connected by a 0.1 mm strip-line to contact pads (Figure 1a-c). The electrodes were thermally evaporated on an Edwards High Vacuum 4P system onto a thermally oxidized Si wafer (SiO_2_), used as substrate. The surface was first cleaned for 15 min using sonification with acetone and isopropanol at room temperature, followed by drying with N_2_. Using a stainless-steel shadow mask with a 0.25 mm thickness, a layer of 10 nm of titanium (Ti), followed by 70 nm of gold (Au), was deposited onto the surface of the substrate. Finally, the sensor was ultrasonically cleaned for 10 min in Milli-Q water and isopropanol and exposed to an oxygen plasma treatment for another 10 min, immediately before the SAM activation process.

**Figure 1:**
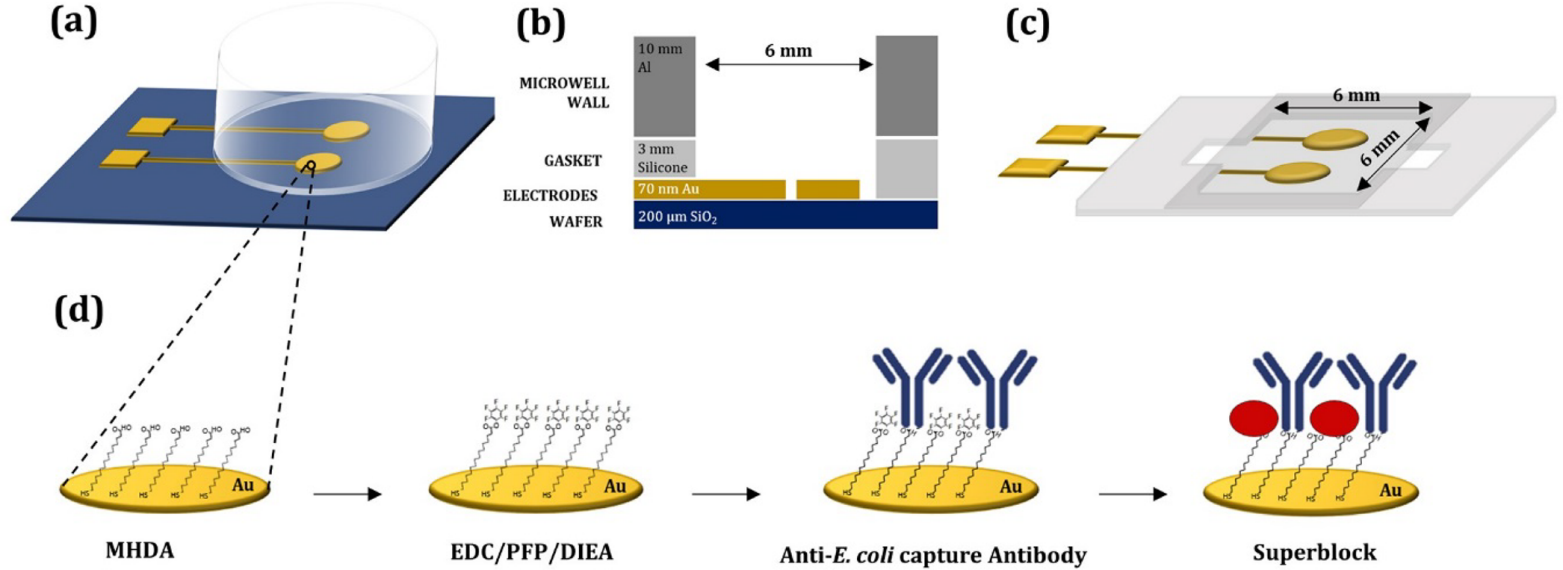
Schematic view of the sensor fabrication: (a) sensor representation of one of the wells with two circular electrodes, each with 1.3 mm^2^ of area. (b) Cross-sectional view of the sensing apparatus, comprising the following layers: SiO_2_ (blue), gold electrodes (yellow), silicone gasket (light gray) and aluminum wall (dark gray). (c) Sensor representation with a PVC microchannel 6 mm (width) × 6 mm (length) × 0.3 mm (height). (d) COOH-terminated self-assembled monolayer (SAM) of mercaptohexadecanoic acid, activation of the surface, antibody incubation, and blocking.

### SAM Activation

Gold functionalization was adapted from previous studies.^4^ Functionalization started by immersing the clean electrodes in 2 mM of MHDA ethanolic solution overnight at 4 °C. Then, the electrodes were rinsed with absolute ethanol and incubated with a combination of EDC (0.2 M), PFP (0.2 M), and DIEA (0.2 M) in absolute ethanol at room temperature (20 °C). After 30 min of incubation, the well was rinsed with absolute ethanol and dried with N_2_.

### EIS and Antibody Functionalization

EIS was recorded at zero applied bias using an EmStat4S potentiostat (PalmSens, Houten, Netherlands) from 10 Hz to 10^5^ Hz, with an AC voltage of 10 mV. The two electrodes were placed within a 6 mm diameter well manufactured out of aluminum and a silicone gasket and connected with a stripline to the external contact pad (Figure 1 a-b). Given the large electrode area, the stripline area was dismissed in all calculations. Data acquisition and modeling were accomplished using PSTrace 5.9 software (PalmSens, Netherlands). All functionalization steps and measurements were carried out at room temperature (20 °C). Real-time EIS measurements were conducted in sets of experiments described further in the text, in the presence of 5 mM [Fe­(CN)_6_]^3–/4–^ in 1× PBS, used as a measuring buffer.

### Effect of Antibodies Concentration on EIS Measurements

In order to analyze the impact of different concentrations in the EIS measurements, the SAM-coated gold electrodes were first incubated with 200 μL of a range of 0–15 μg/mL rabbit polyclonal anti- capture antibodies diluted in 1× PBS buffer. Each concentration was incubated for 2 h at room temperature (20 °C).

### EIS Measurements of Kinetics of Antibody Binding

In order to validate the ability of our technique to track real-time binding of antibodies to the functionalized gold electrode’s surface, we carried out a set of EIS measurements, starting with the addition of 200 μL of 1 μg/mL rabbit polyclonal anti- capture antibodies diluted in measuring buffer and incubated for 20 h. EIS measurements were taken at regular intervals, every 5 min.

### Effect of Diffusion Distance and Surface Area on EIS Signal

To assess how diffusion distance affects antibody binding, we fabricated ’open microchannels’ made out of thin 0.1 mm thick PVC sheets, measuring 6 × 6 mm with 0.3 mm spacing in height (Figures 1c and S4). Similar to the aluminum wells, we positioned these microchannels by centering the pair of gold electrodes. The microchannels, with an internal volume of approximately 30 μL, were sealed by sandwiching an additional layer of PVC that had been previously immersed in Superblock overnight. Experiments were then performed for 1–1000 mM of [Fe­(CN)_6_]^3–/4–^ in 1× PBS (up to 30 min) and for incubations with 1 to 100 μg/mL of anti- capture antibody for 2 h at room temperature (20 °C).

### Fluorescence Microscopy

After immobilizing 1 μg/mL anti- capture antibody for 20 h, the electrodes were incubated with 100 μL of FITC-labeled goat antirabbit IgG secondary antibody (1 μg/mL) for 2 h in the dark at room temperature (20 °C). The functionalized gold surfaces were treated with SuperBlock for 2 h, washed with PBS-Tween-20, and maintained in 1× PBS and 99% glycerol (1:9) at 4 °C. Immunofluorescence was performed using a Zeiss LSM880 Multiphoton Confocal Laser Scanning Microscope (ZEISS, UK) to detect the presence of anti- capture antibody on the surface and to control for nonspecific binding.

### FTIR Characterization of the Gold Surface

After immobilization of the anti- capture antibody, the surface of the electrodes was stored in 1× PBS and analyzed by a Nicolet iS50 FTIR Spectrometer (ThermoFisher, UK), equipped with an MCT/A-based detector. Transmission FTIR spectra were recorded in the wavenumber range of 650–4000 cm^–1^ with a wavenumber resolution of 8 cm^–1^ for 128 scans.

## Results and Discussion

### EIS Offers Real-Time Quantitation of Chemisorption to SAM

Previous studies have used EIS to characterize the binding of a biomolecule onto an activated gold surface or the quantification of antibody–antigen binding at the endpoint once equilibrium is established. In this study, we conducted a systematic analysis of EIS for real-time tracking and quantitation of chemisorption of diagnostic antibodies to a self-assembled monolayer (SAM) on gold electrodes in the presence of a redox probe, 5 mM [Fe­(CN)_6_]^3–/4–^. Real-time monitoring of IgG binding to biosensing surfaces has so far been mostly restricted to advanced optical techniques like SPR, yet this is important for the development of any biosensing device involving a biofunctionalized surface, as both the orientation and extent of binding impact the performance of the biosensor.^1^ Consequently, we recorded impedance and phase angle spectra for a range of frequencies between 1 and 10^5^ Hz, for increasing concentrations of IgG antibody ranging from 0 to 15 μg/mL (Figure 2a-c), in the presence of 5 mM [Fe­(CN)_6_]^3–/4–^ in 1× PBS buffer, and in a 6 mm internal diameter well matching the design of a standard 96 microwell plate. We noticed significant changes in impedance as the antibody concentration increased (Figure 2a,c). At low frequencies (<10 Hz), impedance values increased (Figure 2a) from 4.48 × 10^4^ to 1.80 × 10^5^ Ω, and capacitance decreased (Figure 2d) from 8.47 × 10^–8^ to 4.91 × 10^–8^ F with increasing IgG concentration, suggesting that the increase in low-frequency impedance was mostly due to a decrease in capacitance. The relaxation frequency, *f*_r_, which describes the dispersion in capacitance obtained from the maximum in the corresponding dielectric loss, increased as a function of IgG concentration (Figure 2d). The dielectric loss is characterized as the equivalent parallel conductance, *Gp*, over the angular frequency, ω. The dispersion captured by the relaxation frequency, *f*_r_ is given by eq 1, as in previous work:^6,19^

1 $$f_{r}=\frac{1}{2\pi }{\left(Q\frac{R_{\mathrm{ct}}\times R_{\mathrm{sol}}}{R_{\mathrm{ct}}+R_{\mathrm{sol}}}\right)}^{-\frac{1}{n}}=\frac{1}{2\pi }{\left(QR_{\mathrm{sol}}\right)}^{-\frac{1}{n}}$$

where the parameter *Q* is independent of frequency and holds a phenomenological nature. The charge transfer resistance is given by *R*_ct_, the resistance of the solution is given by *R*_sol_, the Helmholtz double-layer capacitance *C*_dl_, while the exponent *n*, approximately 0.9, aligns closely with the expected value of 1 for an electrode that approaches ideal polarization.

**Figure 2:**
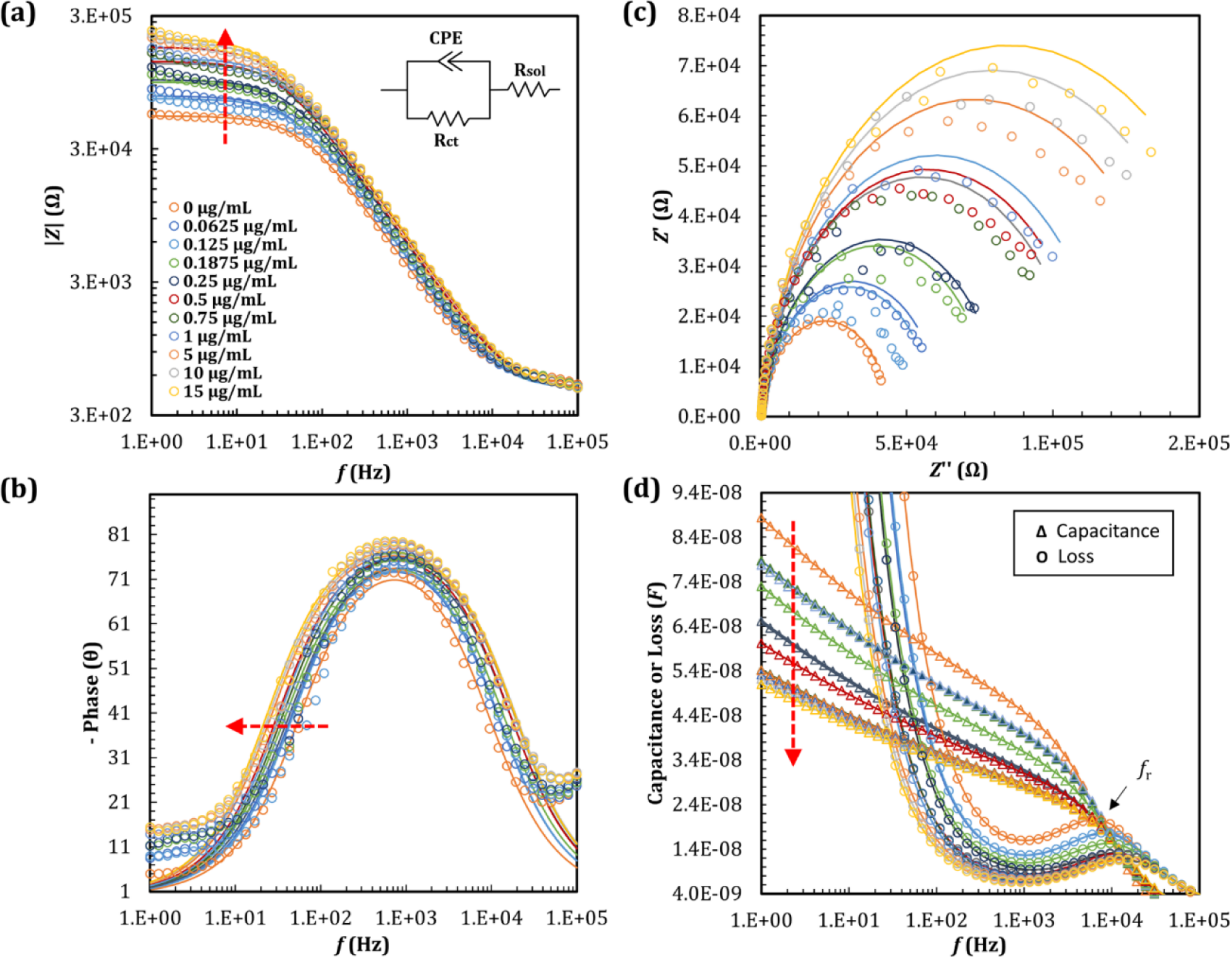
EIS characteristics for SAM-activated electrodes (1.3 mm^2^ area) before and after 2 h of incubation of various concentrations of IgG antibody (0.0625, 0.125, 0.1875, 0.25, 0.5, 0.75, 1, 5, 10, and 15 μg/mL). Experimental (symbols) and fitted (lines) data, measured between 1 Hz and 10^5^ Hz: (a) Impedance (Ω) as a function of frequency (Hz) in logarithmic scale. The Randles equivalent circuit model is represented in the inset. (b) Phase angle (θ) as a function of frequency (Hz) in semilog scale. (c) Nyquist plot, where *Z’’* and *Z’* are the real and imaginary parts of the impedance (Ω), respectively. (d) Capacitance (*F*) and dielectric loss, *Gp*/ω (*F*) over frequency (Hz) in semilog scale. The Maxwell–Wagner relaxation frequency is shown as *f*_r_.

EIS offers several key advantages for the real-time monitoring of antibody adsorption onto SAMs on gold electrodes. Unlike Cyclic Voltammetry (CV), EIS enables a more sensitive and quantitative assessment of interfacial changes associated with biomolecular adsorption in the presence of a redox probe, allowing for the extraction of parameters that are directly influenced by the adsorption and coverage of antibodies on the SAM. These parameters provide a valuable insight into the kinetics and extent of chemisorption, which cannot be easily inferred from peak current variations in CV. While CV peak currents can reflect changes in the electroactive surface area, they are also influenced by mass transport and redox kinetics, making it challenging to isolate adsorption-specific contributions. In contrast, EIS allows for the differentiation between physisorbed and chemisorbed species by modeling impedance spectra with appropriate equivalent circuits, providing a more direct and quantitative approach to assessing chemisorption behavior, as explained in a previous study.^20,21^ Additionally, EIS operates at a small perturbation amplitude, ensuring minimal disruption to the SAM-antibody system, whereas CV, due to its sweeping potential, may introduce perturbations that could affect adsorption equilibrium and SAM integrity.^22^ These factors make EIS a more suitable technique for studying antibody adsorption in real time, preserving the integrity of the system while offering high sensitivity.

The measured impedance data were fitted with the equivalent circuit shown in the inset of Figure 2a, comprising a capacitance described as a Constant Phase Element (CPE), and two resistances, *R*_ct_ and *R*_sol_. The best-fitted parameters were summarized in Table S1. *R*_sol_ predominantly originated from the resistance of the electrolyte solution, including the redox probe, remaining unaffected by modifications taking place directly at the electrode surface. The double-layer capacitance was described by a constant phase element, CPE (inset, Figure 2a), since the measured capacitance was not constant and moderately increased with decreasing frequency, in agreement with previous works that used CPE to reflect possible surface defects and/or variations in its properties.^4,6,20,23,24^ We extracted the double-layer capacitance, *C*_dl_, from CPE using Hsu’s approximation:^25^

$$C_{\mathrm{dl}}=\frac{{\left(QR_{\mathrm{ct}}\right)}^{1/n}}{R_{\mathrm{ct}}}$$ 2

*C*_dl_ values steadily decreased with increasing antibody concentration up to 0.75 μg/mL, representing a drop of 37% comparing to the standard double-layer capacitance (see Table S1). This reduction was generally promoted by the interaction and binding events on the surface.^26^ We have estimated this IgG concentration to be within the range of concentrations expected to yield a half to full surface monolayer on the SAM-functionalized gold electrode, with an IgG mass density of 150–600 ng/cm^2^ for flat-on or end-on orientations against the surface, with parallel Fab (fragment antigen-binding), respectively.^1^ Considering the surface area-to-volume ratio of the well, being 1.7 cm^2^/cm^3^, resulting from 200 μL solution in a 6 mm internal diameter well, this antibody concentration was estimated as yielding a mass density of 441 ng/cm^2^, suggesting that the double-layer capacitance remained constant once a full monolayer was established in the well (see Table S1). These estimates are in close agreement with a previous study by Barreiros dos Santos et al.^4^ that investigated the immobilization of IgG mass density on SAM-modified gold surfaces through SPR measurements, indicating a saturation coverage concentration of approximately 476 ng/cm^2^, by using a factor of 120 m° per 100 ng/cm^2^, considering the shift in angle of minimum reflectivity.^27^ While our equivalent circuit model adequately captures the main electrochemical processes associated with antibody chemisorption and interfacial properties, diffusion-controlled processes may still influence the impedance response at low frequencies and are often modeled using a Warburg element.^28,29^ Here, we did not find significant differences in the fitting accuracy when using the Warburg element and, therefore, opted to not increase circuit complexity and interpretation. Additionally, data-driven approaches such as machine learning (ML) methods, e.g., neural networks, support vector machines, among others, offer the potential to complement traditional modeling by improving parameter extraction, reducing subjectivity, and enabling more robust interpretations.^30^ Although ML techniques were not applied in the present work, their incorporation represents a promising avenue for future research in biosensor characterization and real-time impedance analysis.

We have observed a steady increase in low-frequency impedance values as the antibody concentration increased (Figure S1a). At lower antibody concentrations, the impedance values increased linearly with increasing antibody concentration, similar to a first-order process. Then, above ∼1 μg/mL, it shifted toward a zero-order process, in line with the theory for a Langmuir monolayer equilibrium.^1^ The trend in both absolute (Figure S1a) and relative (Figure 3a) values of impedance closely matched the trends observed for best-fitted *R*_ct_ values summarized in Figures S1b and 3b, suggesting that the chemisorption of antibodies to SAM-activated gold electrodes followed a monolayer equilibrium, in full alignment with previous reports^1^ that used optical techniques.

**Figure 3:**
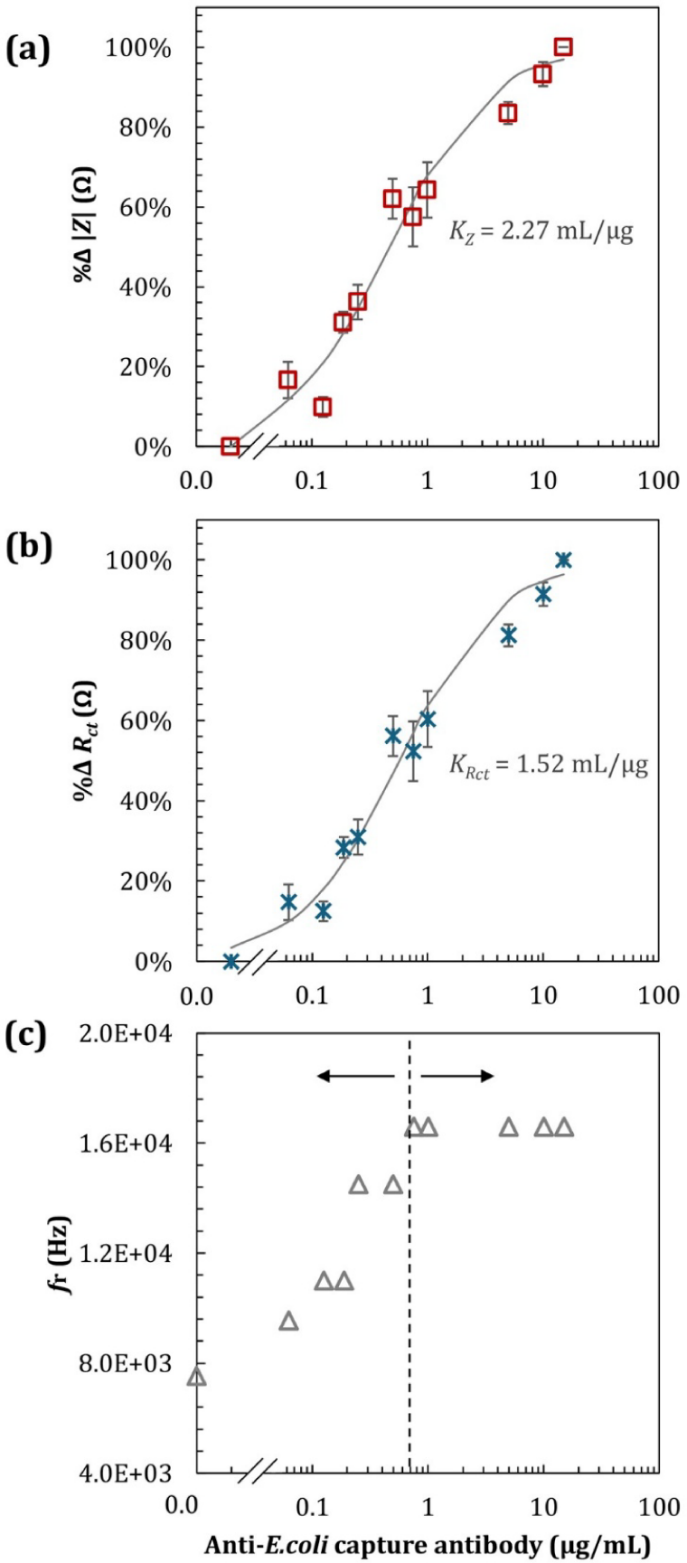
EIS characteristics in semilog scale, for functionalized electrodes, before and after 2 h of incubation of various concentrations of IgG (0.0625, 0.125, 0.1875, 0.25, 0.5, 0.75, 1, 5, 10, and 15 μg/mL). Relationship between (a) %Δ|*Z*| and (b) %Δ*R*_ct_ and antibody concentrations, with the continuous lines showing the best-fit Langmuir isotherms, calculated using eq 3. (c) Maxwell–Wagner relaxation frequency (*f*_r_) over IgG concentration. All error bars shown in (a) and (b) indicate the standard deviation (±1 SD) based on at least 3 experimental replicas, reflecting the reproducibility of the measured impedance parameters.

The charge transfer resistance, *R*_ct_, is sensitive to surface modifications, such as analyte binding, as it interferes with electrode-transfer kinetics at the interface and, consequently, the resistance to electron transfer.^31^ The antibody binding process limits the redox probe’s ([Fe­(CN)_6_]^3–/4–^) ability to approach and transfer electrons to the electrode surface, which expectedly leads to an increase in *R*_ct_ (Figure S1b). To accommodate small variations in absolute impedance values for each replicate, we have normalized impedance and *R*_ct_ data by plotting the relative percentage change in Δ*Z* and Δ*R*_ct_ (*R*_ct_^IgG‑SAM^ – *R*_ct_^IgG^) as shown in Figure 3a,b, respectively, as a function of antibody concentration. We have consequently modeled the chemisorption of IgG to SAM based on the percentage change in Δ*Z* and Δ*R*_ct_, which closely fitted the Langmuir isotherm summarized in eq 3:

3 $$\tau =\tau_{\max}\frac{K\times \left[\mathrm{IgG}\right]}{1+K\times \left[\mathrm{IgG}\right]}$$

where τ is the surface coverage in equilibrium (ng/cm^2^), *τ*_max_ is the number of binding sites available, given by a maximum binding concentration (ng/cm^2^), *K* is the adsorption constant (mL/μg), and [IgG] is the antibody concentration in solution (μg/mL). Model parameters were best fitted to experimental data using Excel’s solver with minimum square differences, from which we obtained the adsorption constant values, *K*_*Z*_ = 2.27 mL/μg and *K*_*R*ct_ = 1.52 mL/μg, respectively, both showing a good fit with *R* ^2^ = 0.97. Some variability was observed in Δ|*Z*| and Δ|*R*_ct_| at specific concentrations (0.125 and 0.5 μg/mL), showing variabilities of 2% and 5%, respectively, but these points did not qualify as statistical outliers according to the IQR method. The error bars in Figure 3a,b represent the standard deviation (±1 SD) from at least three experimental replicates, indicating the reproducibility of the measurements. Although the Langmuir model has been previously reported to fit a correlation between low concentrations of IgG and the optical signal in microcapillaries,^1^ to our knowledge, this work establishes for the first time a direct correlation between the chemisorption of IgG to SAM and the electrochemical impedance signal in real time, demonstrating EIS as a technique for real-time kinetic tracking and quantitation of equilibrium Langmuir isotherms.

Maxwell–Wagner relaxation is an interfacial relaxation process that occurs in all systems where electrical current travels via an interface between two dielectrics.^6^ Often, this relaxation effect is described by the Randles equivalent electrical circuit,^5,18,19^ as presented in the inset of Figure 2a. These biomolecules can introduce additional dielectric properties and alter the charge distribution at the interface. This effect was also analyzed as a function of antibody concentration (Figure 3c). We have noticed a saturation in *f*_r_ of 1.66 × 10^4^ Hz for antibody concentrations above ∼0.75 μg/mL in the 6 mm i.d. well, in line with observations from Figure 2a,c, suggesting that *f*_r_ is sensitive to antibody orientation and remains steady for higher surface protein density packing. Moreover, the relationship between capacitance and impedance has been shown to be inversely proportional up to half-to-full monolayer (Figure S2) since above ∼1 μg/mL impedance values increased without a significant decrease in capacitance, with an attenuation ratio of approximately 0.63-fold. This suggests that for higher IgG concentrations, the SAM has reached full capacity for chemisorption of IgG molecules. Currently, there is no “gold-standard technique” available to directly confirm the orientation of the antibodies, yet it is widely established in the scientific literature that antibody orientation changes with surface coverage/packing.^1,32^

### Surface Binding to SAM Gold Electrodes in a Well is Governed by Mass Diffusion

With EIS established as a technique for real-time tracking of the chemisorption of IgG to gold electrodes functionalized with SAM, subsequent experiments aimed to demonstrate the possibility of using EIS to capture the mass transfer or molecular diffusion process during the chemisorption process. Consequently, a set of experiments was carried out with 1 μg/mL of IgG, i.e., equivalent to half-to-full monolayer. Real-time impedance measurements were made in the presence of the redox probe to detect antibody binding for up to 20 h. The measured impedance and phase angle increased sharply during the initial 2 h of incubation (Figure 4a,b), with a slope of ∼74 kΩ/h, followed by a slow yet steady increase after ∼4 h of incubation, with a mean slope in |*Z*| of ∼43 kΩ/h. This suggested that chemisorption events of IgG became less frequent beyond the initial 2 h period, which was linked to molecular diffusion. While the variability, represented by the shaded area (±1 SD), increased as the rate of |*Z*| change slowed, the underlying trend remained consistent. It is important to note that this variability reflects the experimental reproducibility over time and is distinct from the quality of the fitting of the equivalent circuit shown in Figure 2.

**Figure 4:**
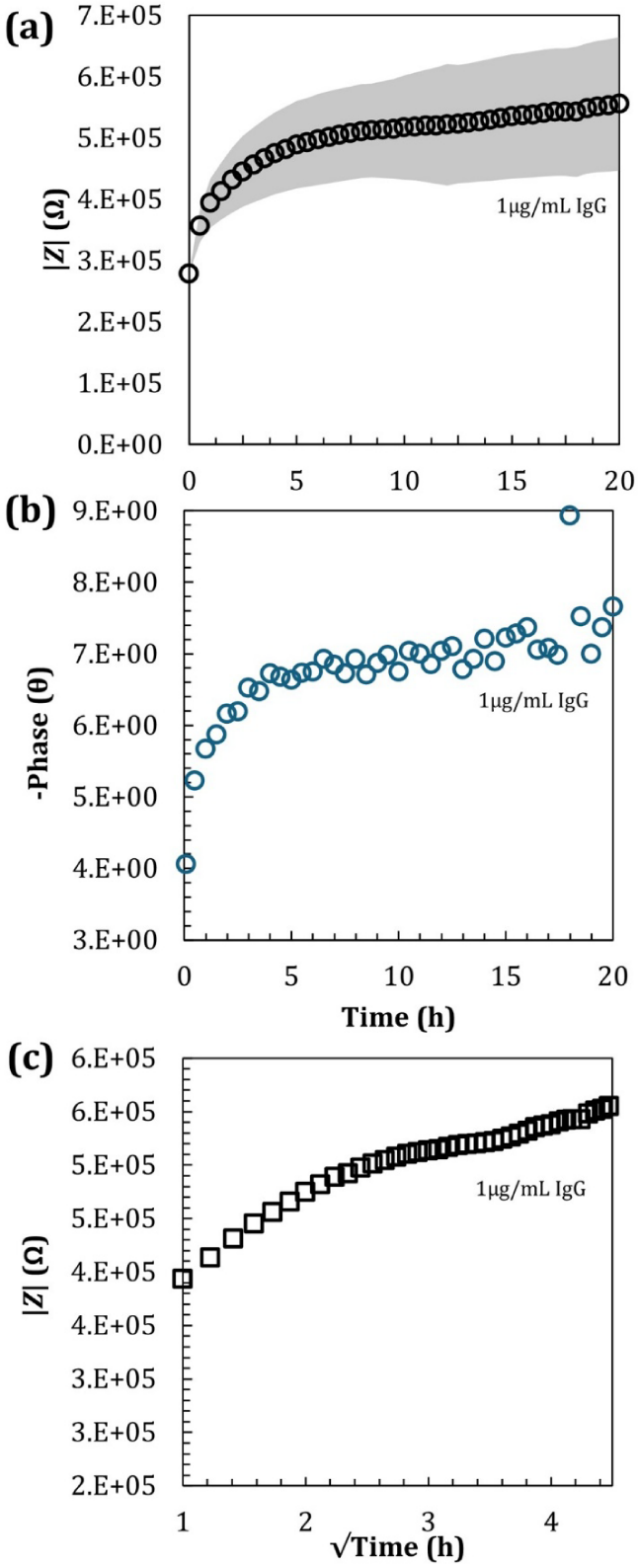
EIS results for the incubation of 1 μg/mL of IgG over 20 h of incubation. (a) Impedance (|*Z*|) and (b) Phase angle as a function of time. (c) Impedance (|*Z*|) as a function of time^1/2^, suggesting the process becomes diffusion-limited beyond the initial 2 h, due to the long distance of diffusion of IgG in the well. The light gray area in (a) represents the standard deviation (±1 SD). This shaded area was used for visual clarity and representation of at least 3 experimental replicas, indicating experimental variability; nonetheless, the general trend remained robust.

A volume of 200 μL in the 6 mm inner diameter well provided a maximum liquid height or a maximum diffusion distance of ∼7 mm. Diffusion time can be predicted based on the Stokes–Einstein law for diffusion^33^ presented in the following equation:

4 $$D=\frac{{\mathrm{\Delta ̅}}^{2}}{2t}$$

where *D* is the diffusion constant and ${\mathrm{\Delta ̅}}^{2}$ is the mean square of the deviation in a given direction over time, *t*. Taking the molecular diffusion coefficient as *D*_IgG_ = 2.81 × 10^–7^ cm^2^/s,^34^ we have estimated a maximum diffusion time of ∼25 h, covering the full extent of the measurements. Figure 4c suggests that beyond the initial 2 h, the binding process is still mostly controlled by the diffusion effect, showing an almost linear signal increase versus *t* ^1/2^.

Previous studies suggested that changes in impedance and electrode properties can be triggered by exposure to the redox probe [Fe­(CN)_6_]^3–/4–^ resulting in degradation of the electrode-monolayer interface.^20^ However, it is important to note that the impact on the SAM’s stability is more noticeable under light exposure.^35^ In our study, all measurements were conducted in dark conditions. During numerous experiments carried out in our study, we did not observe any evidence of degradation of the electrode due to the presence of the redox probe.

### Effect of Diffusion and Surface-to-Volume Ratio

As we miniaturized impedance measurements from a 6 mm wide well holding 200 μL to a 0.3 mm height flat microchannel holding 30 μL, we observed that the electrical response of the SAM-functionalized electrodes changed significantly. We hypothesized that the electrical response is dependent not only on diffusion effects but also on the surface-to-volume ratio and, therefore, the concentration of the probe. This can have a large impact in terms of miniaturizing impedance-based biosensors. Most studies found in the literature report the need to have a redox probe at a significant concentration, usually 3–10 mM,^31,36^ yet miniaturization consequently increases the surface area-to-volume ratio, which suggests that the concentration of the redox probe has to be revised. Consequently, a series of experiments carried out in the well at increasing ion concentrations of 1–100 mM [Fe­(CN)_6_]^3–/4–^ showed gradual *R*_ct_ reduction of up to more than 2 orders of magnitude (Figure 5). This result was expected because of the increased concentration of the redox probe diffusing to the underlying electrode. The maximum diffusion time for the redox probe, based on eq 4 and the diffusion coefficient found in the literature,^37^ *D* = 4.18 × 10^–10^ m^2^/s was ∼1.74 h, which is in agreement with the duration of these experiments (2 h). We noticed that higher concentrations of the redox probe contributed to increased stability in impedance measurements over time (Figure 5a), with data suggesting that 50 mM of [Fe­(CN)_6_]^3–/4–^ yielded good stability and rendered the electrode suitable for observing substantial changes in impedance behavior.

**Figure 5:**
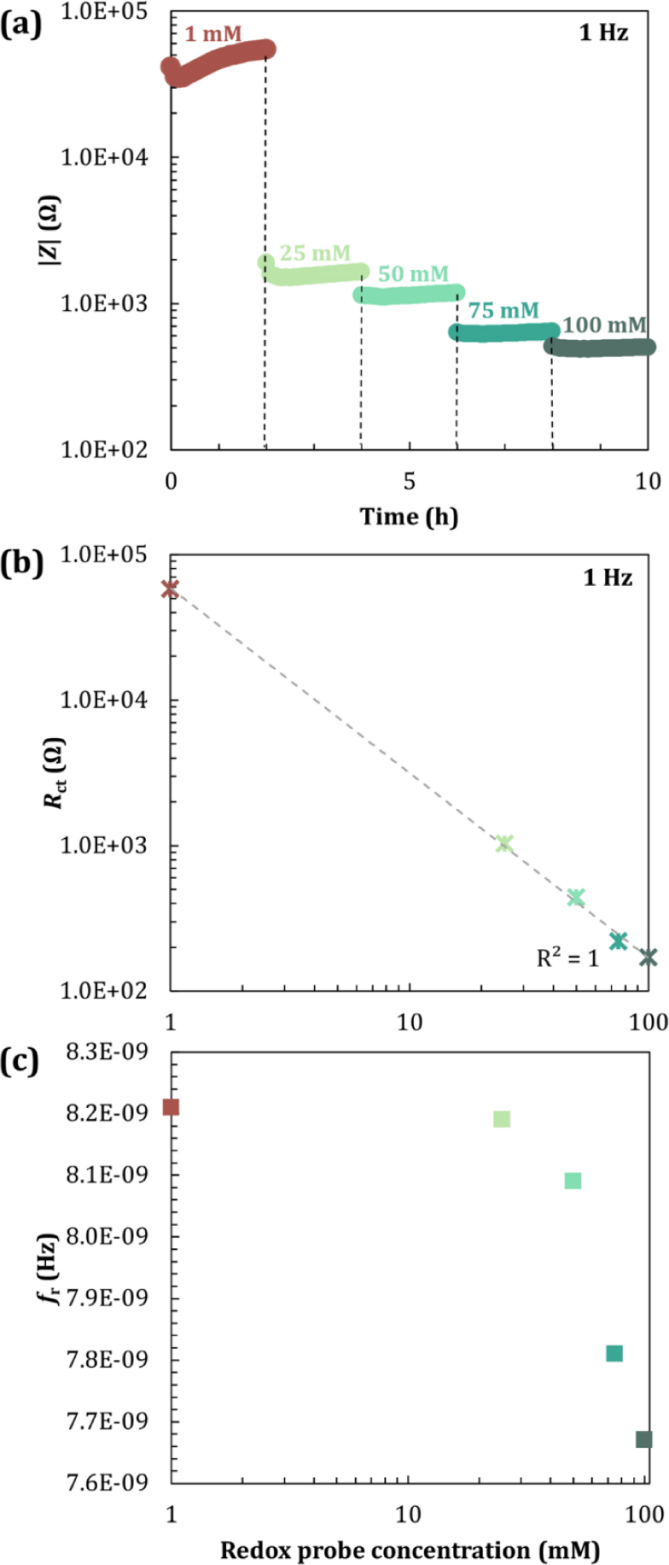
EIS measurements with redox probe concentrations between 1 and 100 mM [Fe­(CN)_6_]^3–/4–^ in 1× PBS in a 6 mm internal diameter well. (a) Impedance values for various redox probe concentrations over 2 h each (at 1 Hz) in semilog scale. (b) Charge transfer resistance as a function of the concentration of redox probe (mM), at *t* = 0, presented in log scale. (c) Maxwell–Wagner relaxation frequency (*f*_r_) over redox probe concentration (*t* = 2 h).

Chemisorption of IgG to the SAM-functionalized gold electrodes is believed to occur through the formation of amide bonds.^4^ The introduction of MHDA results in the presence of a carboxyl group, and its subsequent activation using DIEA, EDC, and PFP leads to the formation of a pentafluorophenyl ester, a highly efficient reactive intermediate for amide bond (Figure 6). Thus, prior to chemisorption of IgG, the surface of the electrode was likely to be primarily charged due to the presence of the reactive intermediates, which could introduce a charge to the surface due to the electron-donating properties associated with the presence of this group and a probably less pronounced polar nature due to the previous introduction of the carboxyl groups. The presence of an electron-donating group may make it capable of interacting with positively charged ions in the surrounding solution. In the case of such interactions occurring, significant interference can potentially affect protein linkage formation, competing with or hindering the reaction with the amino group.

**Figure 6:**
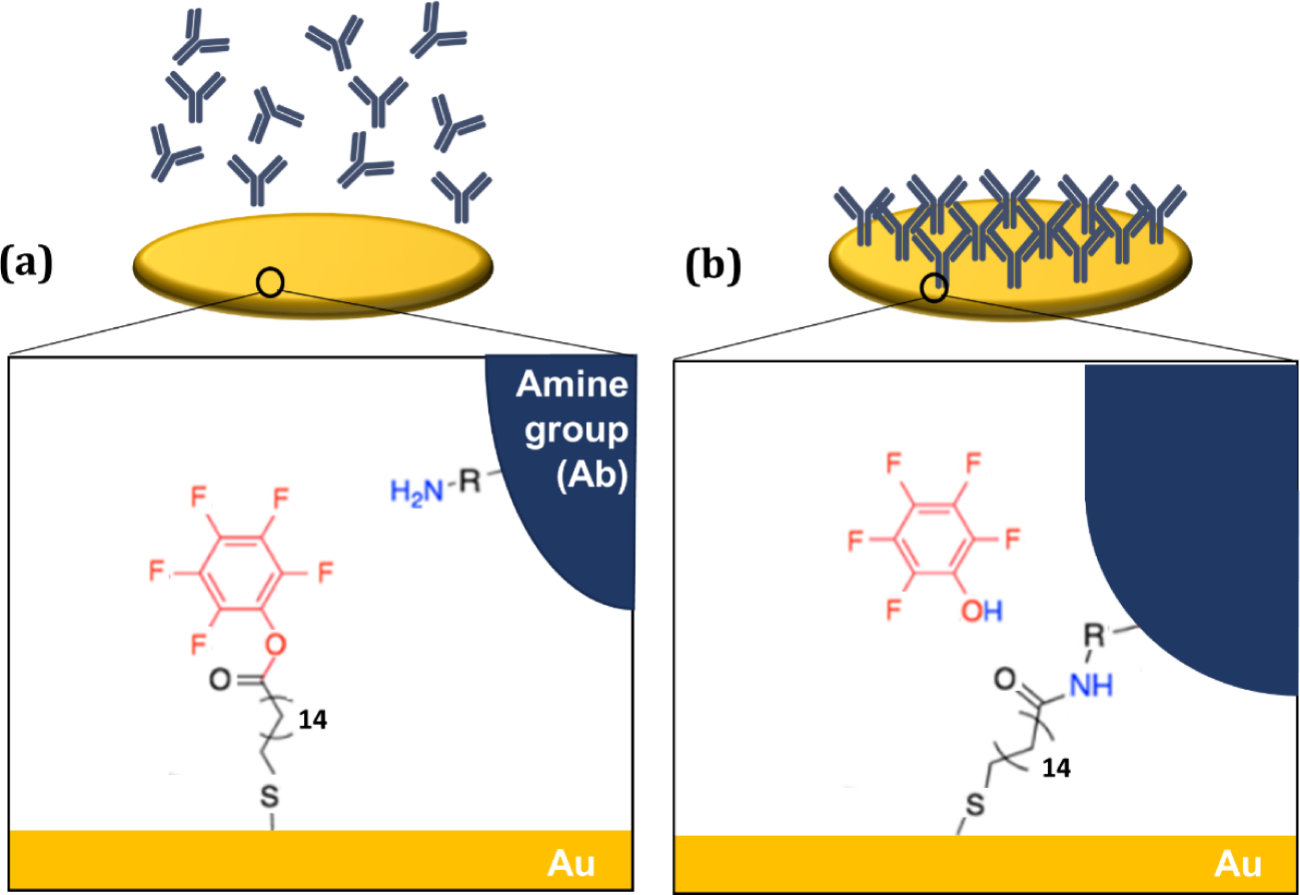
Amide bond formation on gold electrodes. (a) SAM activation, with the surface modified to form pentafluorophenyl ester as a reactive intermediate. (b) Surface after chemisorption of IgG antibody.

By using a flat microchannel with a reduced diffusion distance of 0.3 mm, the maximum diffusion time was estimated through eq 4 as ∼15 s for the redox probe and ∼4 min for the IgG molecule. Initially, the response of bare gold electrodes and SAM-functionalized electrodes was tested with increasing concentrations of 5–500 mM [Fe­(CN)_6_]^3–/4–^ (Figure 7a). We noticed, however, that the impedance signal, in general, did not stabilize within the time scale of the experiments (30 min) for the lower concentrations of the redox probe, suggesting that the impedance measurements were controlled by an equilibrium reaction and potentially by the desorption of a functional group from the surface of the electrode (Figure 7a). We observed a decrease in low-frequency impedance (Figure 7b) and *R*_ct_ (Figure 7c) with the increase in redox probe concentration, similar to the observations in the well (Figure 5b). In Figure 7c, both experimental data plotted on a log scale show an empirical relationship that aligns well with a power regression model, although the underlying mechanisms need further investigation. Comparatively, the *R*_ct_ values obtained in the SAM-activated electrodes were about 2 orders of magnitude higher compared to the bare gold surface, confirming the presence of the antibody monolayer on the surface of the electrodes, which in turn hinders charge’s transfer.

**Figure 7:**
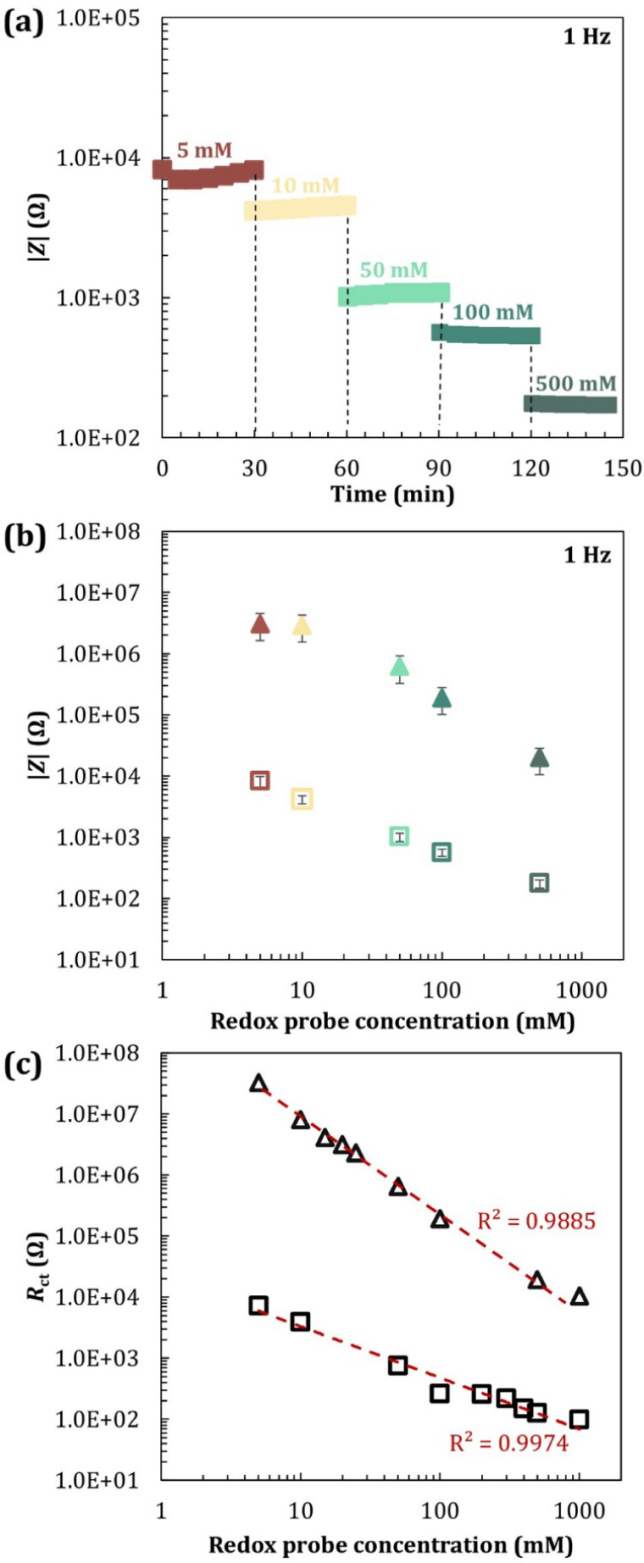
EIS measurements for concentrations between 5 and 500 mM of [Fe­(CN)_6_]^3–/4–^ in 1× PBS (at 1 Hz). Measurements were performed using a flat microchannel with 0.3 mm in height. (a) Impedance values for various redox probe concentrations over 2 h on a bare gold surface, in semilog scale. (b) Impedance values for various redox probe concentrations on bare gold (squares) and activated SAM (triangles). (c) Charge transfer resistance as a function of the concentration of redox probe (mM), presented in log scale, for bare gold (squares) the activated SAM on surface (triangle). All error bars shown in (a) represent the standard deviation (±1 SD) based on at least 3 experimental replicas. Dashlines in (c) show a power regression fitting to the experimental data.

At low concentrations of the redox probe, below 5 mM, the low-frequency modulus at 1 Hz is not capable of accurately translating surface changes (Figure 5a). In contrast, as shown in Figure 7a, the low-frequency modulus for 5 mM or above exhibits relatively closer readouts among distinct molarities, between 10 and 500 mM, and improved electrochemical stability over time. To enhance miniaturized detection with a SAM-functionalized electrode surface, the redox probe concentration was thus increased to above 5 mM. Figure 5a suggests that 50 mM [Fe­(CN)_6_]^3–/4–^ yielded good stability in impedance measurements over time. However, a further set of measurements with SAM-functionalized electrodes at increasing IgG concentrations (Figure 8) in the presence of 50 mM of redox probe in the microchannel yielded electrical behavior distinct from that observed with the well. To infer the potential effect of the redox probe on the stability of SAM or the chemisorption of IgG, we compared measurements carried out in the presence and absence of the redox probe in the IgG buffer. When IgG was incubated with the redox probe added to the buffer, the addition of antibodies resulted in a sharp decrease in the charge transfer resistance, from approximately 101 kΩ to 70 kΩ, showing stabilization from this point at an IgG concentration of 10 μg/mL, which we believe is close to the half-monolayer concentration for the microchannel, based on a mean surface coverage of 300 ng/cm^2^.

**Figure 8:**
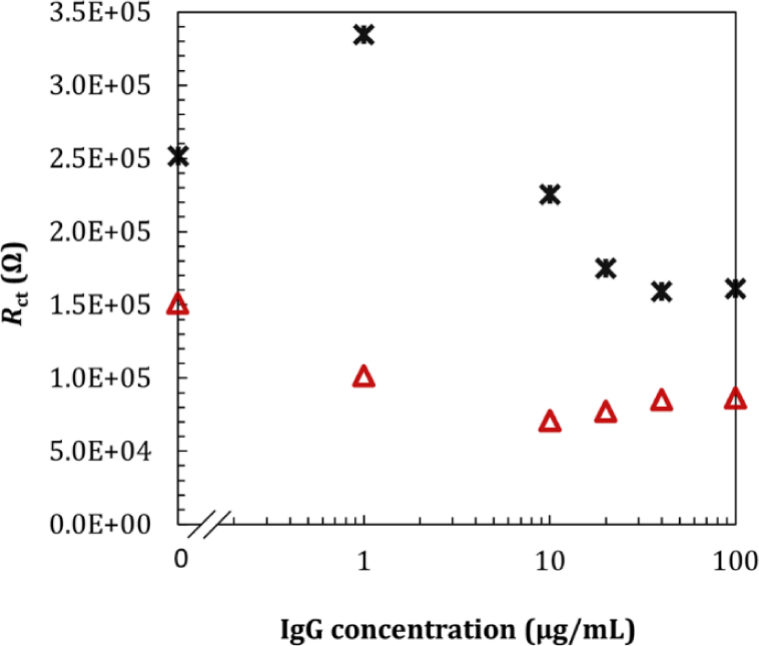
EIS measurements for various IgG concentrations (1, 10, 20, 40, and 100 μg/mL) using 50 mM of [Fe­(CN)_6_]^3–/4–^ in 1× PBS, presented in semilog scale. Measurements were performed using a flat microchannel, height of 0.3 mm. *R*_ct_ for IgG incubated in the presence of redox probe (triangle, red) and *R*_ct_ for IgG incubations in 1× PBS (star, black).

The presence of a high concentration of redox probe in Figure 8 indicates an increase in the concentration of ions in the sample. According to Latour et al.,^38^ and considering the activated SAM as having charged surface chemistry, negatively charged groups can rapidly form electrostatic complexes with positively charged ions, e.g., Na^+^, frequently present in buffers (Figure 9a). Due to the faster diffusion rate of cations and anions from dissociated salts in physiological solutions compared to proteins, the charged functional groups of both the protein and the material surface form complexes with counterions well before the protein’s can diffuse to and interact with the material surface, resulting in fewer peptides adsorbing to the surface (Figure 9b). An increase in ion concentrations in the sample can increase the chances of this interference occurring, which may hinder the antibody binding process and therefore result in a decrease in *R*_ct_. The increase in *R*_ct_ observed under certain conditions is therefore linked to other molecular events, in particular, the orientation and aggregation of IgG onto the surface of the electrode.

**Figure 9:**
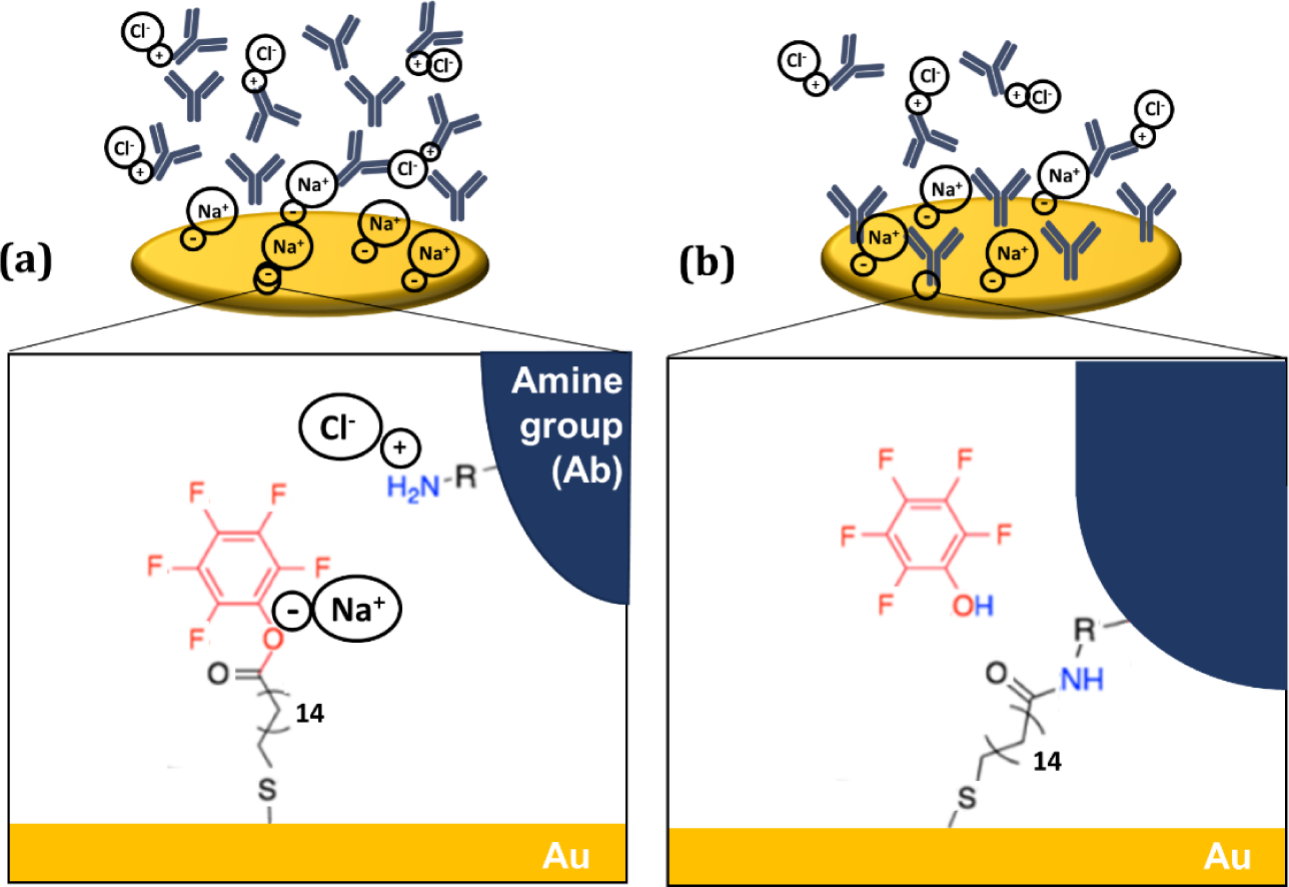
Amide bond formation on gold electrodes and interaction between the charged peptides and the ions in the solution. (a) SAM activation, showing the surface is modified to form pentafluorophenyl ester as a reactive intermediate, interacting with the ions in the solution. (b) Surface showing competitive binding between proteins and ions on buffered solution.

With IgG incubated in 1× PBS and the redox probe added during the measurements, we observed an initial increase in *R*_ct_ up to 1 μg/mL IgG, followed by a sharp decrease with increasing antibody concentrations. These two sets of measurements suggested 2-fold implications. First, chemisorption of IgG molecules at low concentrations, where antibodies are known to adsorb “flat-on”, is potentially disturbed by the presence of a high concentration of the redox probe. Second, the electrical response of the electrode is potentially sensitive not only to the amount of IgG chemisorbed but also to the orientation of the IgG, as we observed a slight increase in *R*_ct_ for IgG concentrations of 20–100 μg/mL, which, in the case of the microchannel, corresponds to a full monolayer and above. Previous works have shown that above a full surface monolayer, there is potential for aggregation of protein on the surface, yielding a reduction in the free energy barrier and changes in protein–protein interactions,^1^ which presumably can be captured with our methodology. Experiments repeated with 100 mM redox probe concentration (Figure S3) showed undetectable differences in the impedance signal.

### Surface Characterization of Functionalized Gold Electrodes

We have confirmed the chemisorption of IgG to the SAM-functionalized gold electrodes using confocal laser scanning microscopy (LSCM) and Fourier transform infrared spectroscopy (FTIR). The LSCM technique allows the demonstration of surface functionalization through fluorescence imaging. An anti-rabbit IgG antibody was used to recognize the capture IgG antibody chemisorbed on the SAM-activated electrode’s surface versus a control surface. As expected, the chemically activated surface (Figure 10a) showed no fluorescence in the absence of the primary antibody, thus not showing other background interferences and nonspecific bindings. On the other hand, Figure 10b shows a specific binding of the secondary antibodies to the immobilized primary capture antibody, thus demonstrating its presence on the activated surface of the electrodes. Nevertheless, the image reveals a sparse fluorescence signal on the gold electrode surface. As we are examining molecules labeled with fluorescent fluorophores, at half-monolayer coverage, the fluorescence signal remains modest, as the concentration of the bound secondary antibody is lower and governed by the affinity constant of the antibodies. Note that FTIR signals shown had the background signal subtracted for bare gold.

**Figure 10:**
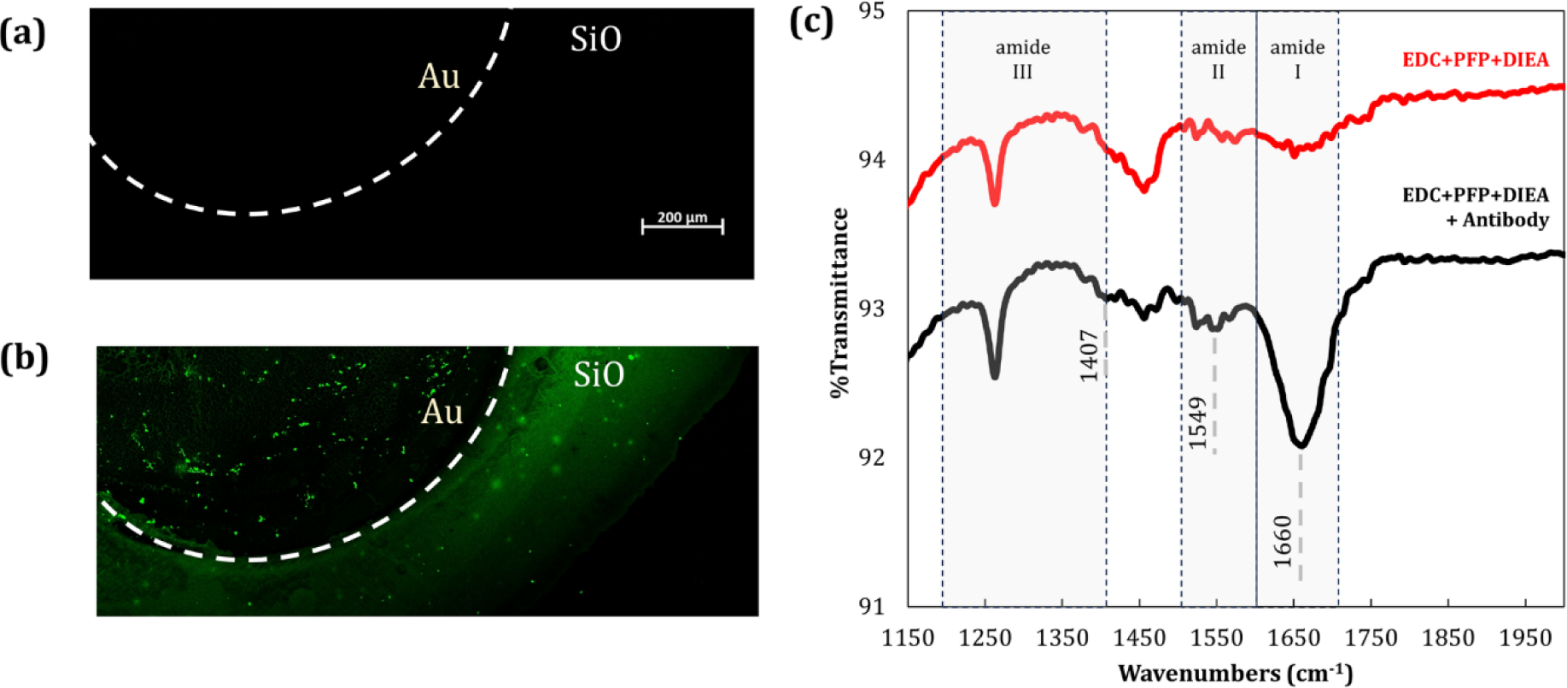
Fluorescence microscopy and FTIR analysis confirming IgG antibodies adhesion to gold electrodes surface. (a) Control for nonspecific binding of antirabbit IgG in the absence of capture IgG antibody. (b) Gold electrode’s surface functionalized with IgG antibody and probed with selective antirabbit IgG. (c) Fourier-transform infrared spectra of chemically activated gold surface, before (red) and after the immobilization of the IgG antibodies (black).

Infrared spectroscopy is a technique largely applied to analyze the structure and interaction of proteins and peptides,^39,40^ especially for secondary structures.^41–45^ Amide I (1600–1700 cm^–1^), amide II (∼1550 cm^–1^), and amide III (1200–1400 cm^–1^) are vibration regions most commonly used for protein structure analysis.^39,44–48^ The analysis between the chemically activated surface before and after the immobilization of antibodies showed an intense peak at 1660 cm^–1^ and another peak at 1549 cm^–1^, falling in the amide I and amide II spectra, respectively. The amide I is primarily due to C=O stretching vibrations, and the amide II band consists of mainly N—H bending but also C—N and C—C stretching vibrations.^49,50^ The capture of a peak within each of the amide I and II spectra is consistent with other studies involving IgG antibody protein,^39,44–46,48,51–53^ including gold-coated surfaces.^54^ A small peak was also captured at 1407 cm^–1^ due to C—OH vibrations, with a similar result previously reported.^55^ A background analysis on the bare gold surface of electrodes was also performed and removed from the data shown for clarity. A small change was also noticeable within the amide III region, although weak and of little use for structure analysis. This could be due to the fact that it is a spectrum of more complexity, with the vibrational region significantly prone to interferences from buffer molecules.^44^

## Conclusions

A systematic study of real-time quantitation of chemisorption of IgG to a large SAM-activated gold electrode surface has been successfully demonstrated using EIS. The impedance spectra recorded for increasing IgG concentrations showed a 3.4-fold increase in impedance at low frequencies (<10 Hz) as antibody concentration increased from 62.5 pg/mL to 20 μg/mL. Dynamics in terms of impedance, *R*_ct_, capacitance, and relaxation frequency all changed as IgG concentration reached the half-to-full monolayer equilibrium in a 6 mm inner diameter well. Modeling of antibody binding based on the percentage increase in impedance and *R*_ct_ closely fitted a Langmuir isotherm, with a shift from first-order to zero-order kinetics as IgG concentration increased, marking, to the best of our knowledge, the first demonstration of EIS as a technique for the direct quantitation of the Langmuir isotherm for chemisorption of antibodies to a SAM. The adsorption constant could be obtained by best-fitting experimental data, establishing a direct correlation between chemisorption and electrochemical signal. The Maxwell–Wagner relaxation effect analysis added further insights, suggesting that *f*_r_ might also be sensitive to antibody orientation, remaining steady at higher surface density. The rate of chemisorption was also found to be dependent on the diffusion distance, as expected for a large molecule like IgG, which theoretically makes miniaturization using, e.g., microchannel compelling. However, the electrochemical response was found to depend not only on diffusion but also on the surface-to-volume ratio. As miniaturization increases the surface-to-volume ratio, a high concentration of redox probe of 50 mM or above was required in order to capture changes in capacitance and impedance, yet with potential interference in the chemisorption of IgG at low concentrations, which subsided around half-to-full IgG monolayer concentration. Fluorescence microscopy and FTIR confirmed successful chemisorption of IgG to the SAM on the gold electrode’s surface. This research establisheds EIS as a potential tool for studying the equilibrium and kinetics of adsorption and chemisorption of biomolecules to electrodes and as a quality-control technique for functionalization of gold electrodes for electrochemical biosensors. Future work will evaluate a wider range of molecules to further validate the scalability and broader applicability of this technique for the manufacturing of SAM-based electrochemical biosensors.

## Supplementary Material



## Notes

Supplementary results, including additional Table S1, with Impedance parameters calculated for various concentrations of IgG; Figures S1 to S3, showing raw data of EIS parameters comparing the incubation of different concentrations of antibody; and Figure S4 showing a schematic of a flat microchannel used for impedance measurements (PDF)

## Abbreviations

IgG: immunoglobulin G
FITC: goat antirabbit IgG (H+L) secondary antibody
SAM: self-assembled monolayer
PVC: polyvinyl chloride
*R*_*sol*_: solution resistance
*C*_*dl*_: double-layer capacitance
*R*_*ct*_: charge-transfer resistance
*f*_*r*_,: relaxation frequency
CPE: constant phase element
MHDA: 16-mercaptohexadecanoic acid
EDC: N-(3-(dimethylamino)­propyl)-N′-ethylcarbodiimide hydrochloride
PFP: 2,3,4,5,6-pentafluorophenol
DIEA: N,N-diisopropylethylamine
SPR: surface plasmon resonance
EIS: electrical impedance spectroscopy
FTIR: Fourier transmission infrared spectroscopy
LSCM: confocal laser scanning microscopy
