# Supplementary material for: Real-Time Monitoring of Chemisorption of Antibodies onto Self-Assembled Monolayers Deposited on Gold Electrodes Using Electrochemical Impedance Spectroscopy
Source: Langmuir. 2025 Jun 17;41(25):15974–86. doi: 10.1021/acs.langmuir.5c01062 (PMC12224300; doi:10.1021/acs.langmuir.5c01062)
Supplement: Supplementary file 1 [file la5c01062_si_001.pdf]

Supplementary information (SI) for:

Real-Time Monitoring of Chemisorption of Antibodies to Self-Assembled Monolayer Deposited on Gold Electrodes using Electrochemical Impedance Spectroscopy

Soraia Oliveira[a,b], Brian V. Jones[c], Pedro Estrela[b,d], Paulo R.F. Rocha[e,*] and Nuno Miguel Reis[a,b,*]

[a] Department of Chemical Engineering, University of Bath, Claverton Down, Bath, BA2 7AY, United Kingdom

[b] Centre for Bioengineering & Biomedical Technologies (CBio), University of Bath, Claverton Down, Bath, BA2 7AY, United Kingdom

[c] Department of Life Sciences, University of Bath, Claverton Down, Bath, BA2 7AY, United Kingdom

[d] Department of Electronic & Electrical Engineering, University of Bath, Claverton Down, Bath, BA2 7AY, United Kingdom

[e] Centre for Functional Ecology-Science for People & the Planet, Associate Laboratory TERRA, Department of Life Sciences, University of Coimbra, Coimbra 3000–456, Portugal

TABLE OF CONTENTS:

Table S1	pp. 2
Figure S1	pp. 3
Figure S2	pp. 4
Figure S3	pp. 5
Figure S4	pp. 6

Table S1. Impedance parameters calculated using the experimentally obtained impedance data from **Figure 2**. The values of surface resistance (R_{ct}*S) and capacitance per unit area have been adjusted by a factor of 2 to accommodate for the utilisation of two identical electrodes during the measurements

Concentration (μg/mL)	Q (Ω^{-1} s^n)	n	R_{ct} (Ω)	R_{ct}*S (Ωcm^2)	C_{dl} ($\mu F/cm^2$)	R_{sol} (Ω)
0.00E+00	1.08E-07	0.909	4.40E+04	4.40E+02	6.3	435.3
6.25E-02	9.57E-08	0.907	6.24E+04	6.24E+02	5.7	403.3
1.25E-01	9.39E-08	0.910	5.96E+04	5.96E+02	5.6	393.0
1.88E-01	8.94E-08	0.902	7.95E+04	7.95E+02	5.2	393.1
2.50E-01	8.00E-08	0.900	8.27E+04	8.27E+02	4.6	379.9
5.00E-01	7.26E-08	0.908	1.14E+05	1.14E+03	4.5	385.7
7.50E-01	6.50E-08	0.911	1.10E+05	1.10E+03	4.0	378.2
1.00E+00	6.34E-08	0.913	1.20E+05	1.20E+03	4.0	379.8
5.00E+00	6.47E-08	0.911	1.46E+05	1.46E+03	4.1	376.3
1.00E+01	6.21E-08	0.913	1.59E+05	1.59E+03	4.0	375.6
1.50E+01	6.03E-08	0.916	1.69E+05	1.69E+03	4.0	381.2

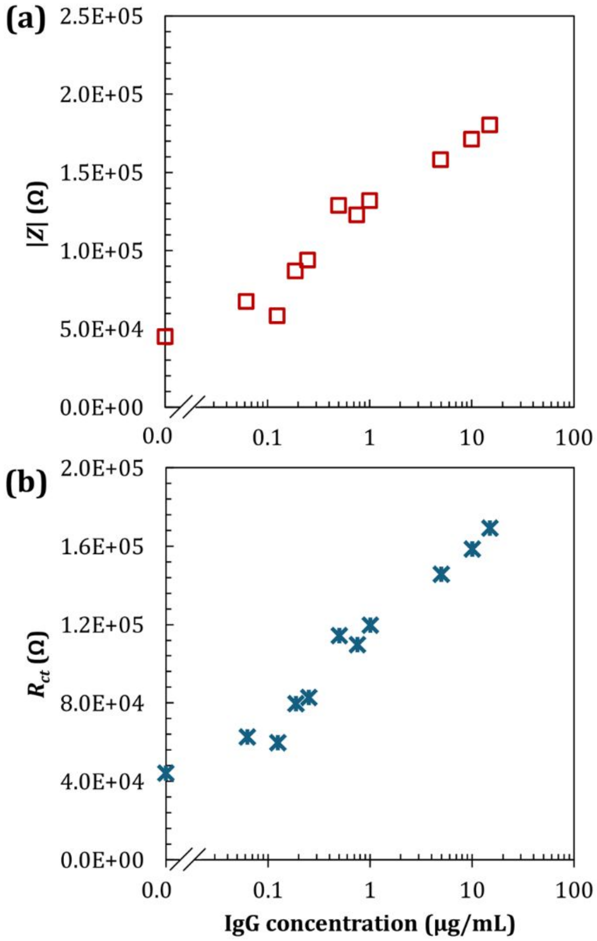

Figure S1. EIS characteristics for functionalised electrodes, before and after 2 h incubation of various concentrations of IgG antibody (0.0625, 0.125, 0.1875, 0.25, 0.5, 0.75, 1, 5, 10 and 15 µg/mL): (a) Modulus of impedance, $|Z|$ and (b) R_{ct} as a function of antibody concentration, at 1 Hz.

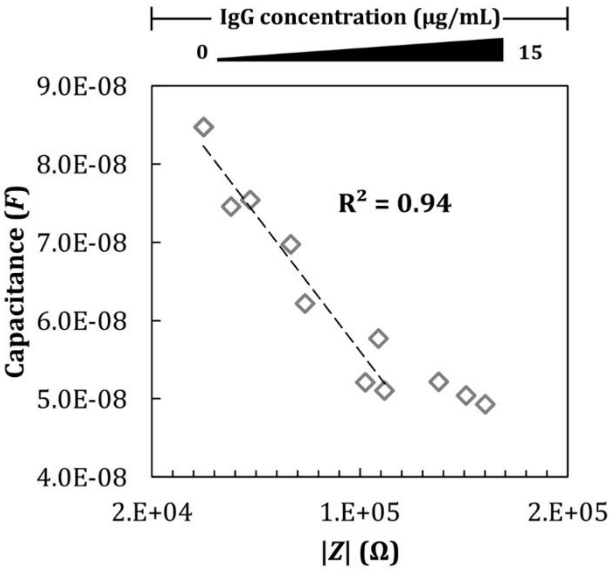

Figure S2. Modulus of impedance, |*Z*| as a function of capacitance, before and after 2 h of incubation of various concentrations of IgG antibody (0.0625, 0.125, 0.1875, 0.25, 0.5, 0.75, 1, 5, 10 and 15 µg/mL), at 1 Hz.

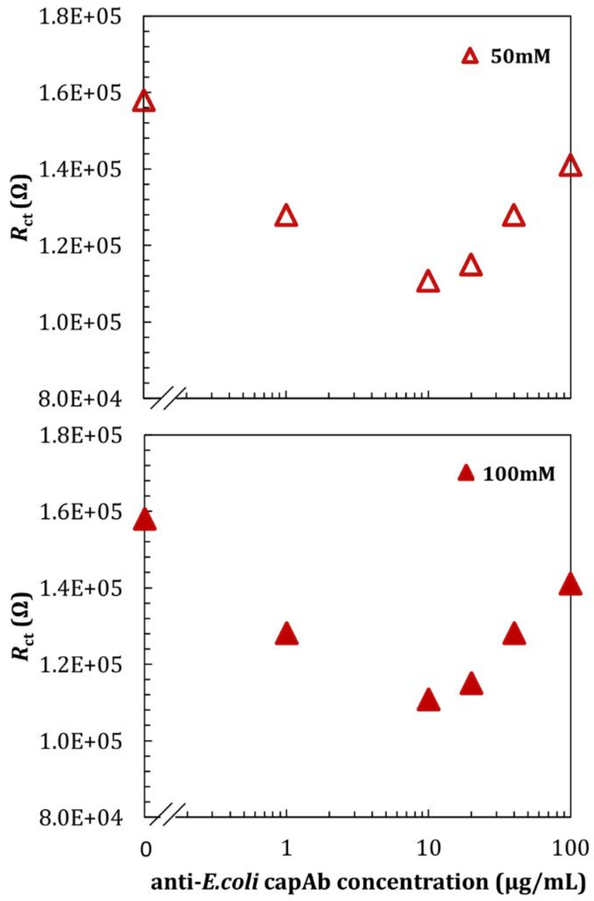

Figure S3. EIS measurements for various antibody concentrations (1, 10, 20, 40 and 100 μg/mL) using 50 mM and 100 mM of $[Fe(CN)_6]^{3-/4-}$ in 1× PBS. Measurements were performed in a flat microchannel shown in Figure S4, having channel height 0.3 mm. R_{ct} over antibody incubations in PBS.

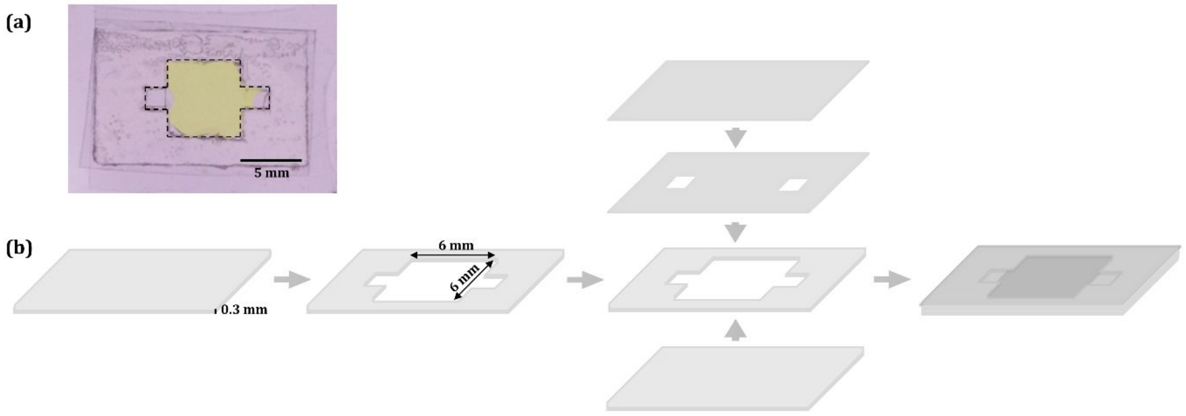

Figure S4. Flat microchannel used for impedance measurements. (a) Photograph of a microchannel, loaded with 30 µl of 5 mM of $[Fe(CN)_6]^{3-/4-}$ in 1× PBS, having internal height of 0.3 mm. The channel was sealed using silicone grease to minimise evaporation. (b) Diagram illustrating the layer-by-layer assembly process used for the fabrication of the microchannels.
